# Expression of Metallic Artifacts Caused by Intracanal Medications with Different Chemical Compositions in Cone Beam Computed Tomography Images

**DOI:** 10.3390/diagnostics15080963

**Published:** 2025-04-10

**Authors:** Giovane Oliveira Silva, Júlia Godoi Lopes, Amanda Pelegrin Candemil, Iago Ramirez, Ruben Pauwels, Manoel Damião Sousa-Neto, Fabiane Carneiro Lopes-Olhê, Giovanni Mergoni, Jardel Francisco Mazzi-Chaves

**Affiliations:** 1 Department of Restorative Dentistry, School of Dentistry of Ribeirão Preto, University of São Paulo, São Paulo 14040-904, Brazil; giovane.14@usp.br (G.O.S.); juliagodoi.jgl@usp.br (J.G.L.); amandacandemil@usp.br (A.P.C.); iagoramirez@usp.br (I.R.); sousanet@forp.usp.br (M.D.S.-N.); fabiane.lopes@usp.br (F.C.L.-O.); 2 Department of Dentistry and Oral Health, Aarhus University, 8000 Aarhus, Denmark; ruben.pauwels@dent.au.dk; 3 Aarhus Institute of Advanced Studies, Aarhus University, 8000 Aarhus, Denmark; 4 Dipartimento di Medicina e Chirurgia, Centro Universitario di Odontoiatria, Università di Parma, 43121 Parma, Italy; giovanni.mergoni@unipr.it

**Keywords:** intracanal medication, root canal treatment, cone-beam computed tomography, X-ray microtomography, artifacts, beam hardening phenomenon, blooming

## Abstract

**Background/Objectives**: Evaluation of artifact expression in CBCT images caused by different intracanal medications (IMs) considering variations in scanning and reconstruction protocols. Reconstruction protocols refer to the specific parameters and image processing techniques applied during CBCT acquisition, including voxel size, slice thickness, and artifact reduction settings. MicroCT was used as the reference standard. **Methods**: Root canal preparation (45./05) of upper canines was performed, and the teeth were divided into four groups (*n* = 10) according to the IM used: G1: Ultracal XS (UC); G2: Bio-C Temp (BCT); G3: Metapex (MT); and G4: Metapaste (MP). The specimens were sealed with temporary provisional material and stored at 37 °C and 100% humidity for 7 days. Then, they were scanned using microCT (SkyScan 1174) and two CBCTs in high and low resolutions: EagleX3D and OP300. Image registration was performed using FIJI ImageJ software (v. 1.54k). Axial, sagittal, and coronal reconstructions were quali-quantitatively evaluated by two calibrated examiners following the scores for the artifacts (dark streaks, hypodense areas, and distortion): definitely absent; probably absent; not sure; probably present and definitely present; and the possibility of using the images for endodontic diagnosis: No/Yes. Statistical analysis was performed using Fleiss’ kappa test and two-way ANOVA (α = 95%). **Results**: CBCT images showed greater volume distortion of intracanal medication (*p* < 0.05) compared to microCT images. X3D CBCT showed the highest values of distortion, regardless of resolution, compared to OP300 (*p* < 0.05). The highest and lowest volume distortion for intracanal medications was observed in the UC and BCT groups, respectively (*p* < 0.05). **Conclusions**: Intracanal medication generates metallic artifact expression in CBCT images, hampering endodontic diagnosis.

## 1. Introduction

The diagnosis in endodontics should be based on data obtained from the combination of anamnesis and clinical examination. Clinical examination should include maneuvers of high diagnostic value, such as inspection, apical palpation, percussion, and pulp vitality testing [1,2,3]. Additionally, imaging exams such as periapical radiography (PR) and cone beam computed tomography (CBCT) are employed to provide essential information about the teeth, root canal system, and adjacent anatomical structures [2,4,5]. The integration of these data is crucial for establishing a prognosis and defining the treatment plan.

Cone beam computed tomography (CBCT) has become a highly effective tool for diagnostic purposes in dentistry [1,2,3]. Its ability to provide depth in evaluation allows for three-dimensional analysis and eliminates the overlap of surrounding anatomical structures, thereby improving diagnostic accuracy [2,6]. Furthermore, this imaging modality allows for examinations with lower operational costs, reduced exposure, and lower radiation doses depending on the selected parameters [6]. A significant advantage of CBCT over other diagnostic imaging methods is its dynamic and interactive navigation of the entire volume of interest, along with simultaneous reconstruction of axial, sagittal, coronal, and parasagittal cuts [7,8].

However, CBCT has some disadvantages, such as longer scanning times and higher radiation doses. Like other imaging modalities that use ionizing radiation, its indication must be carefully justified, ensuring that the benefits outweigh the potential risks [8]. CBCT images often present more image artifacts than periapical radiographs, including distortions in the appearance of objects due to the interaction of X-rays with dense materials, which can lead to the overestimation or underestimation of anatomical structures [1,2,7]. Image artifacts are defined as structures in reconstructed images that do not correspond to the actual scanned object. These artifacts result from discrepancies between the mathematical reconstruction process and the physical process of image acquisition or errors within this process [2,6,9]. The primary cause of artifact generation is the polyenergetic nature of the X-ray radiation used in CBCT and microcomputed tomography (microCT). This results in an image distortion phenomenon known as ’beam hardening’, where highly dense materials, such as radiopaque components in intracanal medications, excessively absorb low-energy X-rays, leading to streaks or dark areas in the final image [1,2,9].

The production of image artifacts presents challenges in the clinical environment, mainly because these artifacts can suggest inconclusive and/or inconsistent results, such as mimicking diagnoses of fractures, perforations [6,9], anatomical variations [10], and under- or over-filling. This can lead to inappropriate therapeutic procedures or even tooth extraction [2,11].

The chemical composition of most intracanal medications and filling materials (e.g., gutta-percha and endodontic sealers) directly influences the formation of various types of image artifacts caused by beam hardening due to the inclusion of radiopaque particles in their formulations [12,13]. The main radiopacifying agents in intracanal medications include zinc oxide (ZnO), barium sulfate (BaSO_4_), bismuth oxide (Bi_2_O_3_), zirconium oxide (ZrO_2_), and calcium tungstate (CaWO_4_) [14,15]. Despite advancements in bioceramic-based endodontic sealers and intracanal medications, radiopacifying agents with high density and atomic numbers, such as calcium tungstate, remain common [15]. However, there are currently no studies in the literature that evaluate the formation and expression of image artifacts in CBCT images caused by intracanal medications based on calcium hydroxide or bioceramic compounds.

Intracanal medications are fundamental adjuncts in endodontic therapy, primarily used to eliminate residual microorganisms, modulate inflammatory responses, and promote periapical healing. Calcium hydroxide-based and bioceramic formulations have demonstrated antimicrobial efficacy and the ability to induce mineralized tissue formation, directly influencing treatment outcomes. However, despite their therapeutic benefits, the high atomic number elements incorporated as radiopacifiers in these formulations may induce image artifacts in cone beam computed tomography (CBCT) scans. These artifacts can compromise diagnostic accuracy by mimicking or obscuring clinically significant structures, potentially leading to misdiagnoses or suboptimal clinical decisions. Given the increasing reliance on CBCT for endodontic assessment, it is essential to investigate the impact of intracanal medicaments on image quality to enhance diagnostic precision and avoid unnecessary interventions.

CBCT imaging, unlike conventional periapical radiography, enables three-dimensional visualization of dental structures, minimizing anatomical superimposition. However, it is subject to various artifacts, particularly beam hardening effects, which result from X-ray attenuation differences between tissues and high-density materials. These artifacts can obscure diagnostic details, particularly in endodontic cases involving radiopaque intracanal materials [1,2,7,9].

The objective of the present study was to evaluate the expression of beam hardening artifacts in CBCT images generated by different scanning parameters and reconstruction protocols. Reconstruction protocols involve specific computational adjustments applied to CBCT scans, including voxel resolution, slice thickness, and metal artifact reduction algorithms. MicroCT was used as the reference standard.

## 2. Materials and Methods

**Figure 1:**
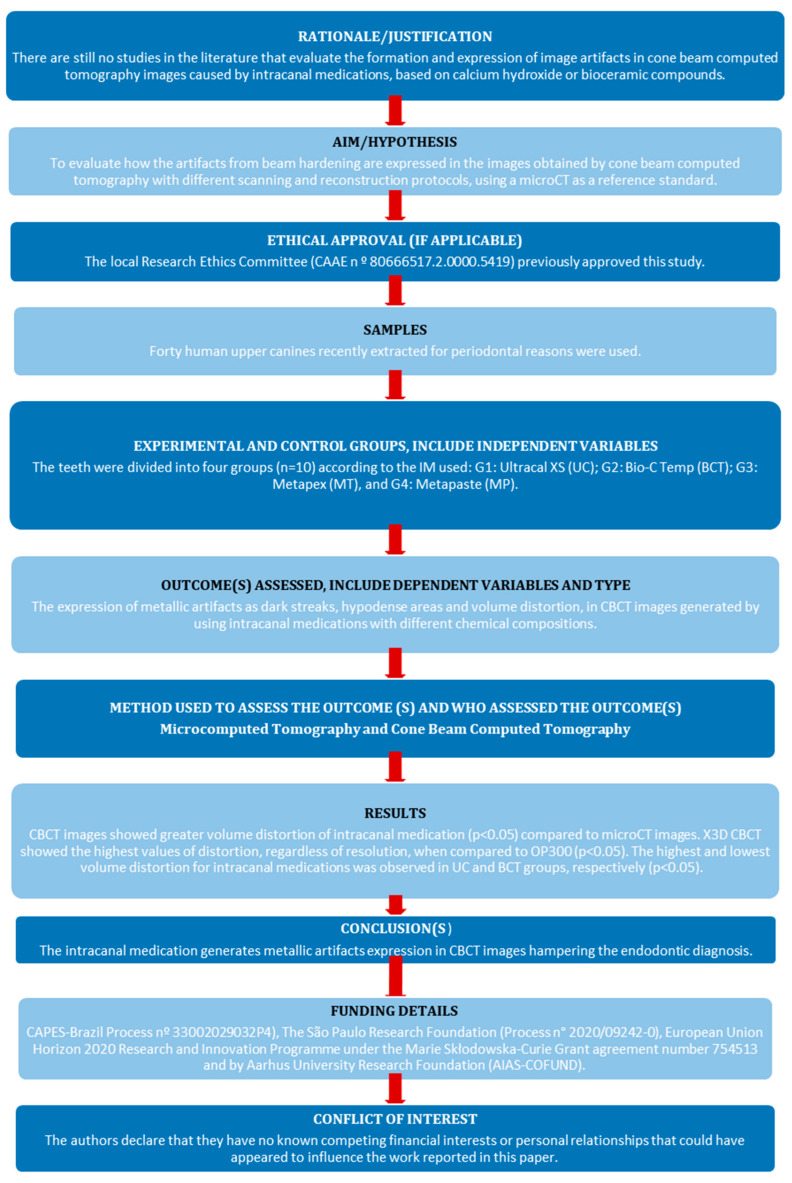
Preferred Reporting Items for Laboratory Studies in Endodontology (PRILE 2021) flowchart.

The manuscript of this laboratory study was written according to the Preferred Reporting Items for Laboratory Studies in Endodontology (PRILE) 2021 guidelines (Figure 1).

The local Research Ethics Committee (CAAE no. 80666517.2.0000.5419) approved this study. Forty human upper canines, recently extracted for periodontal reasons, were used. The teeth were kept in a 0.1% thymol solution until use and were washed in running water for 24 h prior to use. The external root surfaces were cleaned by ultrasonic scaling (Profi II Ceramic, Dabi Atlante Ltd.a, Ribeirão Preto, SP, Brazil) and examined using digital radiography (Spectro 70X Electronic, Dabi Atlante, Ribeirão Preto, São Paulo, Brazil; Fona CDRelite, Schick, DMM, Bandeirantes, PR, Brazil) to pre-select teeth with fully formed roots, closed apices, and canals without calcification, resorption, cracks, or previous endodontic treatment.

After sample selection, the root canals were irrigated with 2 mL of 2.5% sodium hypochlorite (NaOCl) (Fisher Scientific Company, Ottawa, ON, Canada) using a disposable plastic syringe (Ultradent Products Inc., South Jordan, UT, USA) and a 0.30 mm diameter NaviTip needle (Ultradent Products Inc., South Jordan, UT, USA). Root canals were passively explored with #15 K files (Dentsply Sirona Endodontics, Ballaigues, Switzerland) to establish the working length. Biomechanical preparation was performed using the reciprocating motion with the WaveOne^®^ Gold Large instrument (45.05) in pecking movements (Dentsply Maillefer, Ballaigues, Switzerland) coupled to the X-SmartTM Plus electric motor (Dentsply Maillefer, Ballaigues, Switzerland). Chemical irrigation was performed with 2.5% NaOCl (Fisher Scientific Company, Ottawa, ON, Canada) using a disposable plastic syringe and a NaviTip needle (Ultradent Products Inc., South Jordan, UT, USA). Final irrigation was performed with 2 mL of 15% ethylenediaminetetraacetic acid (EDTA) (Sigma Aldrich BVBA, Overijse, Belgium), followed by final irrigation with 5 mL of 2.5% NaOCl (Fisher Scientific Company, Ottawa, ON, Canada) using the conventional syringe/needle irrigation technique. Canal drying was performed with a capillary tip aspiration cannula (Ultradent Products Inc., South Jordan, UT, USA), followed by the use of WaveOne^®^ Large absorbent paper cones (Dentsply Maillefer, Ballaigues, Switzerland).

The teeth were randomly assigned to four groups (*n* = 10) according to the intracanal medication used:

- Group 1: Specimens were filled with calcium hydroxide-based paste—UltracalTM XSTM (Ultradent Products Inc., South Jordan, UT, USA) (UC).
- Group 2: Specimens were filled with bioceramic-based medication—BioC^®^-Temp (Angelus, Londrina, Paraná, Brazil) (BC).
- Group 3: Specimens were filled with iodoform-associated calcium hydroxide paste—Metapex (MetaBioMed, Cheongju-si, Chungcheongbuk-do, Republic of Korea) (MT).
- Group 4: Specimens were filled with calcium hydroxide paste associated with barium sulfate—Metapaste (MetaBioMed, Cheongju-si, Chungcheongbuk-do, Republic of Korea) (MP).

All specimens were filled until medication leakage through the foramen was visible using the manufacturer’s syringe and tip. After filling, the final cleaning of the root canal entrance was performed with an alcohol-moistened sponge, and the specimens were sealed with Coltosol temporary provisional material (Coltène/Whaledent S.A.R.L.). The apex of each root was sealed with nail polish, and the specimens were stored individually in numbered Eppendorf tubes in an incubator set at 37 °C and 100% humidity. The specimens remained in these conditions for 7 days to simulate the clinical conditions of endodontic treatment.

The specimens were scanned using the 1174 Microcomputed Tomograph (Bruker—microCT, Kontich, Belgium). Each specimen was individually positioned and fixed to a metal support with a portion of wax (Boxing Wax, Kerr Corporation, Orange, CA, USA), perpendicular to the radiation source during scanning. The scanning parameters were set at 50 kV, 800 µA, an isotropic resolution of 13 µm, 360° rotation around the vertical axis with a 0.7° rotation step, a total of 3 frames, and a 0.5 mm thick aluminum filter. The scan time was approximately 120 min. The two-dimensional projections generated were archived in Tagged Image File (.tiff) format. After image acquisition, the specimens were returned to the Eppendorf tubes with saline solution and stored in an incubator at 37 °C.

Axial reconstructions were performed from the two-dimensional projections obtained using the NRecon v 1.6.6.0 program (Bruker—microCT, Kontich, Belgium) with a modified Feldkamp cone-beam reconstruction algorithm. Three corrections were applied: Ring Artifact reduction set at a value of 5 (scale 0–20), Beam Hardening set at 50% (scale 0–100%), and Smoothing set at a value of 4 (scale 0–10). The histogram of the images was adjusted on a contrast scale ranging from 0.004 (minimum value) to 0.07 (maximum value), based on previous studies [2]. The reconstructed axial sections were saved in Joint Bitmap (.bmp) format.

For CBCT scanning, the samples were fixed in a macerated human mandible. To simulate soft tissue attenuation during CBCT scanning, the mandible was coated with a 2 cm layer of a mixture called Mix-D, composed of 304 g paraffin, 152 g polyethylene, 32 g magnesium oxide, and 12 g titanium dioxide [1,2,16]. After coating the human mandible with Mix-D, the specimens were individually positioned in artificially enlarged alveoli. The specimens were scanned using two CT scanner devices: Eagle X3D (Dabi Atlante, Ribeirão Preto, São Paulo, Brazil) and OrthoPantomograph OP 300 (Instrumentarium Dental, Tuusula, Finland). The scanning parameters were standardized according to the manufacturer, and two scanning protocols were used: “High Resolution (HR)” and “Standard Resolution (ST)”. The selected field of view (FOV) was the smallest possible provided by the manufacturers (5 × 5 cm). The scanning parameters for high resolution and standard resolution are described in Table 1.

After acquisition, the images were registered using the Elastix program (http://elastix.isi.uu.nl/, accessed on 1 October 2024, University Medical Center Utrecht and collaborators) [1,2,17]. This program is partially based on the Insight Segmentation and Registration Toolkit (ITK). A multi-resolution registration was performed based on the mutual information metric, with the microCT scans as the reference image and the CBCT scans as the “moving” image. The transformation of the recorded image was rigid, allowing only translation and rotation of the image (no scaling changes, which were performed during pre-registration, or other deformations). The aligned CBCT images were saved in .dicom format and converted to .bmp for further analysis.

Two calibrated examiners, one endodontist and one radiologist, performed the qualitative and quantitative evaluations. Both have experience in manipulating and diagnosing from imaging, considering artifact types (dark streaks, hypodense areas, and distortion), scanning protocols (high and standard resolution), and reconstructions (axial, sagittal, and coronal). The evaluations were conducted using ImageJ software v. 1.54k (National Institutes of Health, Bethesda, MD, USA). Reconstructions were selected from all microCT and the two CBCT samples, considering the axial, sagittal, and coronal sections. The same position for each section was used throughout the analysis.

The sections were grouped according to the reconstructions into

1. Axial view
2. Sagittal view
3. Coronal view

They were randomly arranged on different slides in PowerPoint (Microsoft, Seattle, WA, USA), forming an image bank for blinded evaluation. A computer program (https://www.random.org/lists, accessed on 14 October 2024) was used to generate a randomized sequence of images for each sample, which was presented on the same screen using PowerPoint (Microsoft).

Intra- and inter-examiner agreements were assessed using the Fleiss kappa agreement test for each variable under analysis (high resolution or standard resolution, equipment, type of reconstruction, and type of artifact). The results were 1 for the intra-examiner and 0.96 for the inter-examiner, both considered perfect agreements. The qualitative and quantitative evaluations were performed using a personal computer with a screen resolution of 1920 × 1080 (Inspiron 15″ 7568; Dell Computadores do Brasil; Eldorado do Sul, RS, Brazil). To enhance the identification of artifacts generated due to the presence of high-density material inside the root canal system [2,18], brightness and contrast adjustments of the images were performed.

The following types of image artifacts generated by the X-ray beam hardening phenomenon were evaluated:

1. Dark streaks: presence of dark lines observed in the axial reconstructions.
2. Hypodense areas: dark areas or bands adjacent to the high-density material.
3. Distortion: distortion of the volume of the high-density material, also known as the “blooming artifact”. This type of artifact can be observed in all reconstructions [2].

The examiners were instructed to rate the presence and types of artifacts using the following scores [2]:

1. Artifact definitely absent
2. Artifact probably absent
3. Not sure
4. Artifact probably present
5. Artifact definitely present

The images were also evaluated for their quality for endodontic diagnostic purposes after the artifact evaluation. This evaluation parameter involved qualitatively verifying the observation of fractures, cracks, perforations, internal and external resorption, unerupted areas within the root canals, and the presence of fractured instruments. To assess quality for endodontic diagnostic purposes, the images were rated according to two scores: 0 (No) and 1 (Yes).

Subsequently, the observers evaluated the volumes acquired in three-dimensional form through the dynamic and interactive scanning of all images. For this analysis, the multiplanar views (axial, sagittal, and coronal) were adjusted according to the long axis of each root. During this analysis, certain parameters were adjusted to improve visualization, such as magnification, brightness adjustment, contrast, and image thickness. A single examiner evaluated the volumetric distortion of the different medications in the various imaging protocols. The medication volume data obtained from CBCT and microCT images were analyzed using the CTAn program (Bruker, Kontich, Belgium).

The data were statistically evaluated using GraphPad Prism 8.0 software (GraphPad Software, La Jolla, CA, USA). For the evaluation of volumetric distortion, data were subjected to a two-factor ANOVA followed by Tukey’s post-test (α = 0.05), using microCT volume values as reference values. Sensitivity, specificity, and the area under the ROC curve (AUC) were calculated, and variables (materials and devices) were compared using two-way ANOVA and Tukey’s post hoc test at a 5% significance level (*p* < 0.05).

## 3. Results

Table 2 summarizes the volumetric distortion of different intracanal medications across various CBCT acquisition protocols using microCT values as a reference. Accuracy was assessed by the percentage of absolute error (PEA), with thresholding performed by the automatic Otsu method on microCT images. CBCT images exhibited significantly greater volumetric distortion compared to microCT (*p* < 0.05). Among the CBCT devices, Eagle 3D showed the highest distortion values, regardless of resolution, compared to OP300 (*p* < 0.05). The highest volumetric distortion was observed in the UC group, while the BC group showed the lowest distortion (*p* < 0.05).

**Figure 2:**
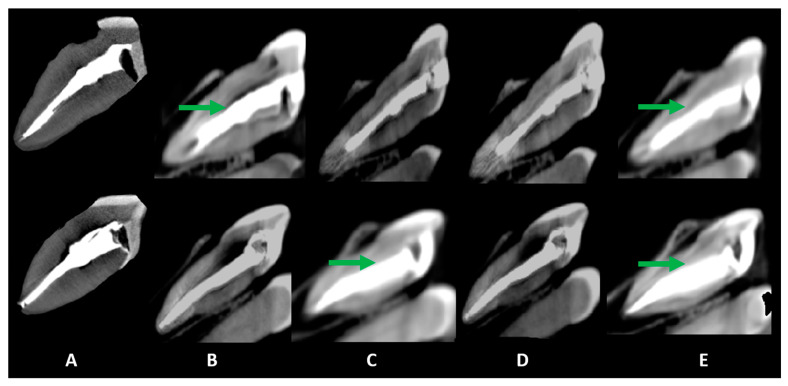
Sagittal slices obtained for the evaluation of artifacts. Volumetric distortion or blooming is indicated by green arrows, showing the actual outline of the filling material. The acquisition protocols are represented by (**A**) micro-CT imaging, (**B**) Eagle 3D HD, (**C**) Eagle 3D ST, (**D**) OP 300 HD, and (**E**) OP 300 ST. A higher expression of dark streaks and hypodense areas can be observed in the images (**B**–**E**).

**Figure 3:**
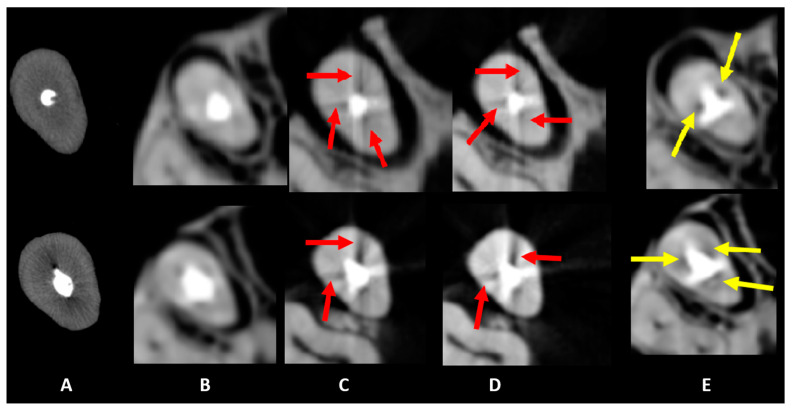
Axial slices obtained for the evaluation of artifacts. Dark streaks and hypodense areas are identified by red and yellow arrows, respectively. The acquisition protocols are represented by (**A**) micro-CT imaging, (**B**) Eagle 3D HD, (**C**) Eagle 3D ST, (**D**) OP 300 HD, and (**E**) OP 300 ST. A higher expression of dark streaks and hypodense areas can be observed in the images (**B**–**E**).

**Figure 4:**
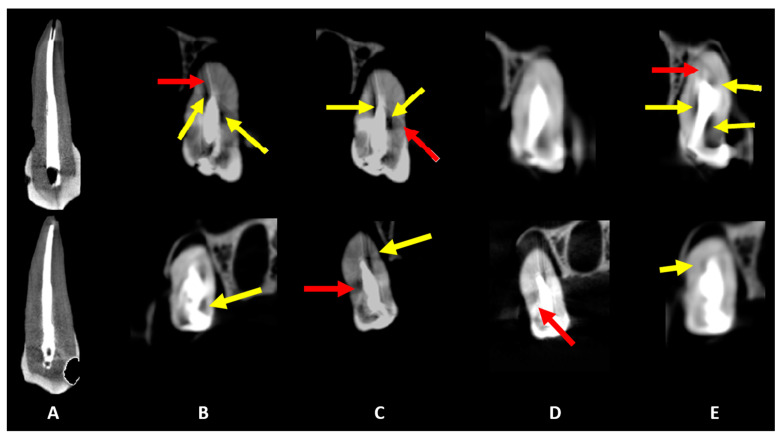
Coronal slices obtained for the evaluation of artifacts. Dark streaks and hypodense areas are identified by red and yellow arrows, respectively. The acquisition protocols are represented by (**A**) micro-CT imaging, (**B**) Eagle 3D HD, (**C**) Eagle 3D ST, (**D**) OP 300 HD, and (**E**) OP 300 ST. A higher expression of dark streaks and hypodense areas can be observed in the images (**B**–**E**).

Figure 2, Figure 3 and Figure 4 illustrate representative examples of different artifact types rather than specific statistical results. The primary data comparisons are presented in Table 2 and Table 3 (Supplementary Table S1).

For hypodense areas, sensitivity, specificity, and AUC were significantly higher in MT and lower in MP for Eagle 3D HR, higher in BC and lower in MP for Eagle 3D ST, higher in BC and MT, and lower in UC for OP300 HR, and higher in MT and lower in UC for OP300 ST (Table 3). Eagle 3D ST (UC and BC) and OP300 HR (MT and MP) showed higher values, while OP300 HR and ST (UC), Eagle 3D ST (MT), and Eagle 3D HR (MP and BC) had lower values (Figure 3 and Figure 4). Detailed statistical values related to sensitivity, specificity, and AUC are provided in Supplementary Table S1. This table presents comparative values across acquisition protocols for different intracanal medications. Regarding volumetric distortion, sensitivity, specificity, and AUC values were significantly higher in UC and lower in MT, BC, and MP for Eagle 3D HR and ST; higher in UC and MP and lower in MT and BC for OP300 HR; and higher in BC and lower in MT for OP300 ST in most conditions (Supplementary Table S1). Conversely, these variables were higher in OP300 HR and lower in OP300 ST for UC, with no significant differences between devices for other materials (MT, MP, and BC) in most conditions (Figure 2, Figure 3 and Figure 4).

Sensitivity, specificity, and AUC values were statistically higher in Eagle 3D HR (UC and BC) and Eagle 3D ST (MT and MP), but lower in OP300 ST (UC), Eagle 3D HR (MT), OP300 HR (MP), and Eagle 3D ST (BC) (Supplementary Table S1).

For dark streak artifacts, sensitivity, specificity, and AUC were higher in UC and lower in MT and BC for Eagle 3D HR; higher in MT and lower in BC for Eagle 3D ST; higher in UC and MT and lower in MP for OP300 HR; and higher in MT and lower in MP and UC for OP300 ST in most conditions (Supplementary Table S1). BC material showed significantly better values for diagnostic use, but no significant differences were observed between devices for most conditions (Table 3). Observer agreement was confirmed with Fleiss’ kappa values of 1.0 (intra-examiner agreement) and 0.96 (inter-examiner agreement), indicating near-perfect reliability. These values reflect the high consistency of artifact classification in CBCT and microCT images.

## 4. Discussion

This study aimed to evaluate the expression of CBCT artifacts induced by different intracanal medications using microCT as a reference standard. Our findings confirmed the hypothesis that the extent of artifact expression varies depending on the type of intracanal medication and CBCT acquisition parameters. The most pronounced volumetric distortion was observed in Ultracal XS, while Bio-C Temp exhibited the lowest level of artifact expression. Additionally, significant differences were noted between the CBCT devices, with Eagle X3D producing greater artifact intensity than OP300. These findings suggest that high-density materials exacerbate beam hardening effects, impairing diagnostic accuracy. A control group without intracanal filling was not included because the primary objective was to compare the relative impact of different intracanal medications rather than establishing a baseline for artifact expression. However, it is important to acknowledge that CBCT inherently produces some degree of artifact due to beam hardening and scattering, even in the absence of high-density materials. To account for this, microCT was used as the reference standard, as it has superior resolution and lower artifact expression, although it is not entirely artifact-free. The minimal artifacts in microCT result primarily from the interaction of high-density materials with the X-ray beam, but they are significantly reduced due to the system’s higher acquisition parameters and reconstruction algorithms. These findings underscore the critical role of the chemical composition and atomic number of intracanal medications in influencing CBCT image quality, directly impacting diagnostic accuracy. The OP300 HR and ST protocols exhibited lower artifact expression compared to Eagle 3D, highlighting their suitability for clinical endodontic diagnostics, particularly in cases involving high-density intracanal medications [1,2,3].

The findings of this study reinforce the clinical relevance of evaluating artifact expression induced by intracanal medicaments in CBCT imaging. While these materials are intended for temporary use, their influence on diagnostic imaging is significant. Artifacts generated by high-density components in intracanal medications can obscure critical anatomical details, leading to misinterpretations such as false-positive diagnoses of root fractures, perforations, or inadequate obturation. This is particularly relevant in cases requiring precise treatment planning, such as retreatment, apical surgeries, or cases involving anatomical complexities. The present study highlights that the type of intracanal medication used should be carefully considered, not only for its biological properties but also for its potential impact on imaging outcomes. Optimizing CBCT parameters and selecting materials with lower artifact potential could contribute to improving endodontic diagnostic accuracy and clinical decision-making.

Artifacts in CBCT images arise mainly from the beam hardening effect, which is significantly influenced by the high atomic numbers of radiopacifying agents, such as bismuth oxide (Bi_2_O_3_), barium sulfate (BaSO_4_), and zirconium oxide (ZrO_2_). These materials are frequently used in intracanal medications due to their high radiopacity; however, they also contribute to pronounced dark streaks and hypodense areas that can obscure important diagnostic details. Bismuth oxide, with an atomic number of 83, produces intense dark streaks and hypodense artifacts, particularly in axial reconstructions, complicating the assessment of root canal anatomy [4,5,6].

Previous studies have demonstrated that high-density intracanal materials generate significant CBCT artifacts, impairing diagnostic accuracy [2,6]. The present findings corroborate these observations, particularly in the case of Ultracal XS and Metapaste, which exhibited pronounced volumetric distortion and dark streak artifacts. These artifacts could potentially obscure fine anatomical details, such as root fractures or accessory canals, leading to misinterpretations in clinical practice.

Calcium hydroxide-based pastes, such as Ultracal XS, typically show fewer artifacts due to their lower atomic number components compared to bismuth or barium-containing medications. The presence of barium sulfate, with its atomic number of 56, significantly increases the formation of hypodense areas and streak artifacts due to its high density, leading to substantial beam hardening effects that distort the anatomical representation of the root canal [7,8,9].

MicroCT was used as the reference standard in this study, displaying minimal artifact formation compared to CBCT due to its superior imaging parameters, such as higher kVp, mA, and longer scan times, which help mitigate the beam hardening effects. Additionally, the smaller voxel sizes and 360-degree rotation contribute to reduced artifacts, offering clearer, more accurate images that are ideal for research, although less practical for clinical settings due to high radiation doses [6,11,12]. The absence of high-density intracanal medications during scanning further reduces artifact expression, emphasizing the need to consider material composition when interpreting CBCT images.

The iodoform component in Metapex, which has a lower atomic number but is combined with calcium hydroxide, results in distinct volumetric distortion and blooming artifacts. These artifacts exaggerate the apparent volume of the filling material, leading to potential overestimation of the root canal space and misleading the clinician in treatment planning [10,13,15]. These findings suggest that CBCT protocols must be carefully selected according to the specific chemical composition of the intracanal medication to minimize diagnostic errors caused by artifacts.

Bioceramic-based medications like Bio-C Temp exhibited fewer artifacts compared to traditional bismuth- and barium-containing materials, likely due to their use of zirconium oxide, which, despite having a high atomic number (40), generates fewer streak artifacts due to better scattering properties. These properties make bioceramics a more favorable option for reducing imaging artifacts, although streaks and hypodense areas can still be observed to a lesser extent [19,20,21]. This highlights the importance of understanding the interactions between CBCT imaging parameters and the specific materials used in intracanal medications.

The differences observed between CBCT devices can be attributed to variations in voxel resolution, exposure time, and artifact reduction algorithms. Previous studies have shown that smaller voxel sizes and higher kVp settings reduce artifact intensity, which aligns with our finding that OP300 produces fewer artifacts than Eagle X3D [12,18]. Moreover, our results reinforce that radiopacifying agents significantly impact CBCT image quality, consistent with prior evaluations of bioceramic-based sealers [10,14]. These findings highlight the necessity of selecting optimized imaging parameters to improve diagnostic accuracy in endodontics.

The study also showed that devices operating at lower kVp settings, such as Eagle X3D, are more susceptible to artifact formation, particularly when scanning materials with high atomic numbers. The increased beam hardening associated with these settings produces darker streaks and more pronounced hypodense areas, compromising image quality. Adjusting imaging parameters such as kVp, mA, and algorithms of post-processing of CBCT images can help reduce the severity of these artifacts, but these adjustments must be weighed against increased patient radiation exposure [2,22,23,24].

Given the influence of different intracanal medication compositions on artifact formation, clinicians need to recognize and account for these effects when interpreting CBCT images. Understanding the specific artifacts produced by various CBCT devices and protocols allows clinicians to optimize imaging settings [22,23,24], enhancing diagnostic accuracy and reducing the risk of incorrect clinical decisions. This is particularly crucial in complex endodontic cases, where clear visualization of the root canal anatomy is vital for effective treatment [2,6,25,26].

Future research should focus on advancing CBCT technology, especially in developing new software algorithms that can effectively reduce the artifacts associated with high-density intracanal medications. Enhancing image-processing capabilities may significantly improve the reliability of CBCT in clinical practice, making it a more dependable tool for endodontic diagnosis and treatment planning [22,27,28]. Further studies should also explore the impact of novel low-density radiopacifiers that could maintain radiopacity while minimizing artifact formation.

Despite the strengths of this study, including a comprehensive comparison of CBCT protocols with microCT, it is important to note the in vitro nature of the research, which may not fully replicate clinical scenarios where tissue attenuation and complex anatomical structures can further affect artifact formation. Future studies should include in vivo assessments to validate these findings and explore the impact of different intracanal medication compositions and atomic numbers on artifact formation. Such research will be instrumental in refining CBCT protocols to ensure optimal image quality and diagnostic efficacy in endodontic practice [22,23,24,29,30].

The clinical implications of these findings are particularly relevant for detecting vertical root fractures, perforations, or residual infection in root-filled teeth. Artifacts from high-density materials, particularly dark streaks and volumetric distortion, can obscure fracture lines, leading to false-negative or false-positive diagnoses. This is especially concerning given that CBCT is frequently recommended for evaluating post-treatment endodontic complications [21,26]. Future research should focus on optimizing CBCT acquisition protocols and developing artifact-reduction algorithms to enhance diagnostic reliability in clinical endodontics.

Intracanal medications with high-density components generate substantial CBCT artifacts, which may compromise diagnostic interpretation. The findings of this study highlight the need for improved CBCT acquisition strategies and artifact reduction techniques. Further research should explore novel radiopacifying agents and post-processing algorithms to enhance CBCT imaging quality in endodontics.

Although this study provided a comprehensive evaluation of CBCT artifact expression related to intracanal medications, a more detailed analysis of image quality parameters such as spatial resolution, contrast resolution, and noise levels, was not performed. These factors are crucial in determining the overall diagnostic reliability of CBCT imaging, as they influence the visualization of fine anatomical details, such as root fractures or accessory canals. Future studies should incorporate these parameters to further refine our understanding of how artifacts interact with CBCT image quality and clinical interpretation.

## 5. Conclusions

This study demonstrated that the expression of CBCT artifacts varies significantly depending on the composition of intracanal medications and the scanning protocol used. Among the materials tested, Ultracal XS exhibited the greatest volumetric distortion, whereas Bio-C Temp produced the lowest artifact expression. Differences between CBCT systems were also observed, with Eagle X3D showing greater artifact intensity compared to OP300, highlighting the influence of imaging parameters on artifact formation. These findings emphasize the need for careful selection of intracanal medications and optimized CBCT protocols to minimize image distortion and enhance diagnostic accuracy. Future research should focus on developing artifact-reduction techniques and optimizing CBCT acquisition settings to improve the reliability of endodontic imaging.

## Figures and Tables

**Table 1:** Descriptive table of the scanning protocols used for the acquisition of images in microCT and cone beam computed tomography.

	MicroCT	Eagle X3D		OP 300	
Field of view (FOV)	-	5 × 5 cm		5 × 5 cm	
Current	800 µA	8 mA		5 mA	8 mA
Voltage	50 kVp	85 kVp		90 kVp	
Isotropic voxel size	13 µm	0.08 mm		0.085 mm	0.20 mm
Exposure time	9000 s	32 s	20.5 s	17.5 s	10.96 s
Filter	Al 0.5 mm	-		-	
Rotation step	0.7°	-		-	
Rotation	360°	360°	360°	360°	360°
Twodimensional projections	516	924	525	870	234
Dose/area product	-	7.42 mGy.cm^2^	4.75 mGy.cm^2^	3.77 mGy.cm^2^	1.62 mGy.cm^2^
Milliamperage/time product	-	154 mAs	87.5 mAs	43.5 mAs	18.7 mAs
Protocol	HR	HR	NR	HR	NR

HR: high resolution; NR: standard resolution.

**Table 2:** Mean and standard deviation of the absolute error of the volumetric distortion for each device and different intracanal medications, with the volume values obtained in microCT as reference.

	Eagle 3D HD	Eagle 3D ST	OP300 HD	OP 300 ST	Total
UC	1.62 ± 0.6 Aa	2.1 ± 0.7 Aab	1.33 ± 0.5 Aba	1.06 ± 0.59 Ba	1.52 ± 0.5 a
MT	0.38 ± 0.08 Aab	0.49 ± 0.1 Aab	0.37 ± 0.11 Aa	0.48 ± 0.54 Ba	0.32 ± 0.7 a
MP	0.64 ± 0.8 Aa	0.6 ± 0.08 Aa	−0.07 ± 0.05 Bab	−0.05 ± 0.07 Bb	0.28 ± 0.05 a
BC	0.15 ± 0.13 Ab	0.22 ± 0.67 Ab	−0.1 ± 0.06 Ab	−0.12 ± 0.1 Ab	−0.013 ± 0.04 b
Total	0.704 ± 0.18 A	0.80 ± 0.18 A	0.38 ± 0.14 AB	0.23 ± 0.18 B	

Uppercase letters mean statistical differences between rows, and lowercase letters mean statistical differences between columns (*p* < 0.05). UC: Ultracal XS; MT: Metapaste; MP: Metapex; BC: Bio-C Temp.

**Table 3:** Sensitivity, specificity, and area under the ROC curve (AUC) for different acquisition protocols and intracanal medications, evaluating image quality for endodontic diagnosis.

Acquisition Protocol		Intracanal Medication			
		UC	MT	MP	BC
Eagle 3D HR	Sensitivity	0 Bb	0.09 Ba	0 Ab	0 Ab
	Specificity	1 Aa	1 Aa	1 Aa	1 Aa
	AUC	0.5 Ab	0.55 Ba	0.5 Ab	0.5 Bb
Eagle 3D ST	Sensitivity	0.3 Aa	0.06 Bb	0 Ac	0 Ac
	Specificity	0 Bb	1 Aa	1 Aa	1 Aa
	AUC	0.014 Bc	0.53 Ba	0.4 Bb	0.5 Ba
OP 300 HR	Sensitivity	0 Bb	0.09 Ba	0.03 Ab	0 Ab
	Specificity	1 Aa	1 Aa	1 Aa	1 Aa
	AUC	0.5 Ab	0.55 Bb	0.52 Ab	0.75 Aa
OP 300 ST	Sensitivity	0 Bb	0.25 Aa	0 Ab	0 Ab
	Specificity	1 Aa	1 Aa	1 Aa	1 Aa
	AUC	0.5 Ac	0.63 Ab	0.5 Ac	0.78 Aa

Uppercase letters mean statistical differences between rows, and lowercase letters mean statistical differences between columns (*p* < 0.05). UC: Ultracal XS; MT: Metapaste; MP: Metapex; BC: Bio-C Temp.

## Data Availability

The data analyzed in the current study are attached, and available from the corresponding author on reasonable request.

## Supplementary Materials

The following supporting information can be downloaded at: https://www.mdpi.com/article/10.3390/diagnostics15080963/s1, Table S1. Sensitivity, specificity, and area under the ROC curve (AUC) values for different acquisition protocols, intracanal medications, and artifact types. MicroCT results were used as the reference standard.
