# Supplementary material for: Expression of Metallic Artifacts Caused by Intracanal Medications with Different Chemical Compositions in Cone Beam Computed Tomography Images
Source: Diagnostics (Basel). 2025 Apr 10;15(8):963. doi: 10.3390/diagnostics15080963 (PMC12025455; doi:10.3390/diagnostics15080963)
Supplement: Supplementary file 1 [file diagnostics-15-00963-s001.zip › diagnostics-3504333-supplementary.pdf]

Supplementary Table S1. Sensitivity, specificity, and area under the ROC curve (AUC) values for different acquisition protocols, intracanal medications, and artifact types. MicroCT results were used as the reference standard.

Aquisition Protocol		Dark streaks				Hypodense areas				Distortions			
		**UC**	**MT**	**MP**	**BC**	**UC**	**MT**	**MP**	**BC**	**UC**	**MT**	**MP**	**BC**
**Eagle 3D HR**	Sensitivity	1 Aa	0.5 Bd	0.73bB	0.67 Ac	0 Ac	0.5 Bb	0 Bc	0.67 Ba	0.67 Ba	0 Ab	0 Bb	0 Ab
	Epecificity	0.36 Ba	0.27 Cb	0.23cB	0.13Dd	0 Cd	0.8 Ba	0.2 Bb	0.13 Dc	0.03 Ba	0.03 Aa	0 Aa	0 Aa
	AUC	0.84 Aa	0.38 Cc	0.49bB	0.38 Ac	0.22 Bc	0.68 Ca	0.1 Dd	0.38 Cb	0.27 Aa	0 Ab	0 Cb	0 Bb
**Eagle 3D ST**	Sensitivity	0.5 Bc	1 Aa	0.55bC	0.11 Cd	0 Ab	0 Cb	0 Bb	1 Aa	0.67 Ba	0 Ab	0 Bb	0 Ab
	Specificity	0.64 Aa	0.36 Ad	0.46bA	0.4 Ac	0.78 Ab	1Aa	0.7Ac	1 Aa	0.6 Aa	0 Ab	0 Ab	0 Ab
	AUC	0.28 Dc	0.68 Aa	0.57bA	0.26 Bc	0.39 Ac	0.5 Db	0.35 Cd	1 Aa	0.12 Ba	0 Ab	0 Cb	0 Bb
**OP 300 HR**	Sensitivity	1 Aa	1 Aa	0.91bA	0.44 Bc	0 Ab	1 Aa	1 Aa	1 Aa	1 Aa	0 Ab	1 Aa	0 Ab
	Specificity	0.32 Ca	0.33 ABa	0cC	0.27 Cb	0.11 Bc	0.4 Db	0 Cd	0.64 Ba	0 Ba	0 Aa	0 Aa	0 Aa
	AUC	0.63 Ba	0.66 Aa	0.23cD	0.36 Ab	0.056 Dc	0.9 Ba	0.7 Ab	0.73 Bb	0.3 Ab	0 Ac	0.56 Ba	0 Bc
**OP 300 ST**	Sensitivity	0.5 Bc	1 Aa	0.91bA	0.44 Bd	0 Ab	1 Aa	1 Aa	1 Aa	0.67 Bb	0 Ac	1 Aa	1 Aa
	Specificity	0.32 Ca	0.32 Ba	0bC	0.33Ba	0.11 Bc	0.5 Cb	0 Cd	0.55 Ca	0 Ba	0 Aa	0.03 Aa	0 Aa
	AUC	0.33 Cc	0.57 Ba	0.32cC	0.39 Ab	0.093 Cd	0.95 Aa	0.5 Bc	0.73 Bb	0.267 Ac	0 Ad	0.64 Ab	0.71 Aa

Uppercase letters mean statistical differences between rows, and lowercase letters mean statistical differences between columns ($p<0.05$). UC: Ultracal XS; MT: Metapaste; MP: Metapex; BCT: Bio-C Temp.
